# Supplementary material for: Pan-genome inversion index reveals evolutionary insights into the subpopulation structure of Asian rice
Source: Nat Commun. 2023 Mar 21;14:1567. doi: 10.1038/s41467-023-37004-y (PMC10030860; doi:10.1038/s41467-023-37004-y)
Supplement: Supplementary file 1 — Supplementary Information [file 41467_2023_37004_MOESM1_ESM.pdf]

Pan-genome inversion index reveals evolutionary insights into the subpopulation structure of Asian rice

Zhou *et al.*

Supplementary Note 1. The 18-genome data package: sequencing, assembly, annotation and results

Sequence and assemblies

The pan-genome of Asian rice includes 16 ultra high-quality genomes that represent the sub-population structure level of the 3K-RGP data set, including: 1) 15 platinum standard reference sequences (PSRefSeqs), and 2) the previously published IRGSP RefSeq[1-3] (Table 1).

In addition, chromosome-level reference assemblies for two wild species - *i.e. O. rufipogon* (AA), and *O. punctata* (BB) were generated using long-read sequencing technology. PacBio subreads were first assembled *de novo* into contigs using genome assembly programs: FALCON (v0.5)[4], MECAT2 (20190314)[5], and Canu1.5[6]. Genome Puzzle Master (GPM, https://github.com/Jianwei-Zhang/LIMS) software[7] was used to merge the *de novo* assembled contigs from the three assemblers, using the MH63 genome sequence as a guide. The assembled genome sequences of *O. rufipogon*, and *O. punctata* were then polished using PacBio long reads and Illumina short reads in SMRTlink6.0 (https://github.com/WenchaoLin/SMRT-Link).

These assemblies have sizes of 461 and 422 Mb, with contig N50s of 32.5 Mb and 33.1 Mb, for *O. rufipogon* (AA), and *O. punctata* (BB) respectively (Table 1 and Supplementary Table 4). Only 7 and 16 gaps remain for the *O. rufipogon* and *O. punctata* genomes (Supplementary Table 4), respectively. Data for these new genomes can be found in Genbank (https://www.ncbi.nlm.nih.gov/) under public BioProjects PRJNA609053 (*O. rufipogon*) and PRJNA13770 (*O. punctata*) (Supplementary Table 4).

BUSCO scores for the two new assemblies were 97.90% (*O. rufipogon*) and 97.00% (*O. punctata*) (Supplementary Table 4). Of note, 16 BUSCO genes were missing in the *O. rufipogon* genome sequence, which were also absent in all *O. sativa* genomes as well[3]. However, 2 (EOG093605AK and EOG09360AWY) out of these 16 genes could be identified in *O. punctata* (BB genome) (Supplementary Table 2). Combined with our previous analysis of Asian rice and maize[3], 14 BUSCO genes are absent in the *Oryza* genus, 12 of which are absent in both the *Oryza* genus and *Zea mays* (Supplementary Table 2).

The assembly and conserved gene content statistics for the *O. rufipogon* and *O. punctata* genomes demonstrate their high-quality, genome-wide contiguity, and completeness. Hence, we regard both assemblies as PSRefSeqs, as previously reported for Asian rice[1-3].

Asian rice pan-genome annotation

To minimize bias associated with different methods of gene and repeat finding, we applied a uniform set of annotation protocols (implemented through MAKER-P[8]) to 16 Asian rice PSRefSeqs (see methods). To accomplish this task, we integrated both Illumina baseline RNA-Seq data (RNA-Seq database#1) with PacBio Iso-Seq data, isolated independently from similar tissues (*i.e.* young leaves, roots, and panicles) (Supplementary Table 1). As a result, we annotated on average 36,347 genes, with an average length 3,448 bp (Supplementary Table 3). These annotations resulted in an expected AED distribution (Supplementary Fig. 1), and BUSCO scores greater than 97.5% for both transcript and protein models (Supplementary Table 4).

Transposable element content

We re-annotated transposable elements (TEs) and investigated TE related sequence (TE-RS) content in the inversions across our 18-genome data set. The overall amount of TE-RSs ranged from 47.41% (*O. sativa XI*-3A LIMA) to 56.54% (*O. rufipogon*) with an average of 51.26% across all 18 genomes (Table 1, Supplementary Data 9).

Supplementary Note 2. Public high-quality rice genome sequences collection and selection

To generate a powerful inversion index for Asian rice, we collected a total of 112 genomes in this study, including the 18-genome data package, and 94 recently sequenced genomes by long-read technologies, *i.e.*, 29 genomes sequenced with PacBio[9] and 65 genomes sequenced with Oxford Nanopore Technology (ONT)[10].

To evaluate each genome assembly, we first compared Contig N50s of all 112 genomes and found that all had Contig N50s > 3 Mb (Supplementary Data 2). However, when all three studies were compared with respect to both Contig N50 size and number of contigs, the 18-genome data package showed the highest overall quality (Supplementary Fig. 3a and 3b).

Next, the 112 genomes were aligned to the IRGSP RefSeq using mummer[11] to look for possible miss assemblies. This analysis revealed that 37 out of 65 ONT genomes had large fragmental translocation (> 5 Mb) that were not validated and were thus filtered out of our analysis. An example of both a retained and filtered genome can be found in Supplementary Fig. 3c and 3d, respectively. The remaining 75 high-quality genomes, *i.e.*, 18-genome data package, 29 new sequenced PacBio genomes and 28 new sequenced ONT genomes, were retained for further investigations.

Supplementary Note 3. Genomic inversion identification workflow

To discover inversions, we first tested four different analysis workflows (see methods) by comparing two *O. sativa* genomes, *i.e.*, *GJ*-temp: IRGSP-1.0 and *XI*-adm: MH63 genomes. We identified 25, 131, 55 and 235 raw inversions based on the 4 workflows, respectively (Supplementary Table 5). Following assessments by dot-plots, 22 (72%), 39 (29.8%), 33 (60%) and 178 (75.7%) raw inversions were confirmed (Supplementary Fig. 4a) for the 4 workflows, respectively, and the false positive ones were filtered out (Supplementary Fig. 4b). In comparison to workflow 4, workflows 1, 2 and 3 identified only 10%, 21.7% and 18.3% of all inversions, respectively (Supplementary Table 5). Upon detailed inspection, we determined that workflow 4 captured the overall majority of true large inversions.

Supplementary Note 4. Machine learning study to measure inversion frequencies across the 3K-RGP data set

To determine the frequency of the inversions detected in the inversion index across the 3K-RGP data set, we trained a machine learning sequential model with 8,720 manually curated alignment patterns (872 inversions across 10 samples). Of note: 80% of these patterns were used for training the model, and another 20% were used for validation. The training model was used to test 30,520 inversion alignment patterns (872 inversions from 35 samples), with a further 5-fold cross validation and correction. The 39,240 manually curated inversion events from 45 samples were then used for training with 5 cross checks, with accuracy reaching > 98% and > 96% for training and validation, respectively (Supplementary Fig. 6c). Five independently trained tests were used to predict inversion events from remaining samples of the 3K-RGP data set, composed of 2,597,688 alignment patterns (872 inversions for 2,979 samples). Three rounds of 2,000 randomly selected events from the uniform results of 5

independent tests were further manually validated, and the results showed accuracies ranging from 94.6% to 100% (Supplementary Table 8).

Based on the observations of Asian rice specific inversions across the 3K-RGP data set, inversion frequencies were studies for each subpopulation. Samples were filtered that had missing data of 30% or greater, and the remaining 631 inversions across 2,751 samples were retained for further analysis (Supplementary Data 7). We used a threshold of observed inversion frequency (OIF) of 0.03 in this study for inversion categories. In doing so, inversions were classified into four groups: 1) genome-specific inversions, *i.e.* the inversions could be observed in only one or only a few (OIF < 0.03) 3K-RGP accessions; 2) subpopulation specific inversions, *i.e.* the inversions could be mainly observed in one of subpopulations but not others (OIF > 0.03); 3) Group-specific inversions, *i.e.* the inversions could be observed in one of groups, *e.g.*, only observed either in *Geng/Janponica* (*GJ)* group, *Xian/Indica* (*XI*) group, *circum-Aus* (*cA*) group, or *circum-Basmati* (*cB*) group genomes, respectively; and 4) shared inversions, *i.e.*, the inversions could be observed in more than one subpopulation or group.

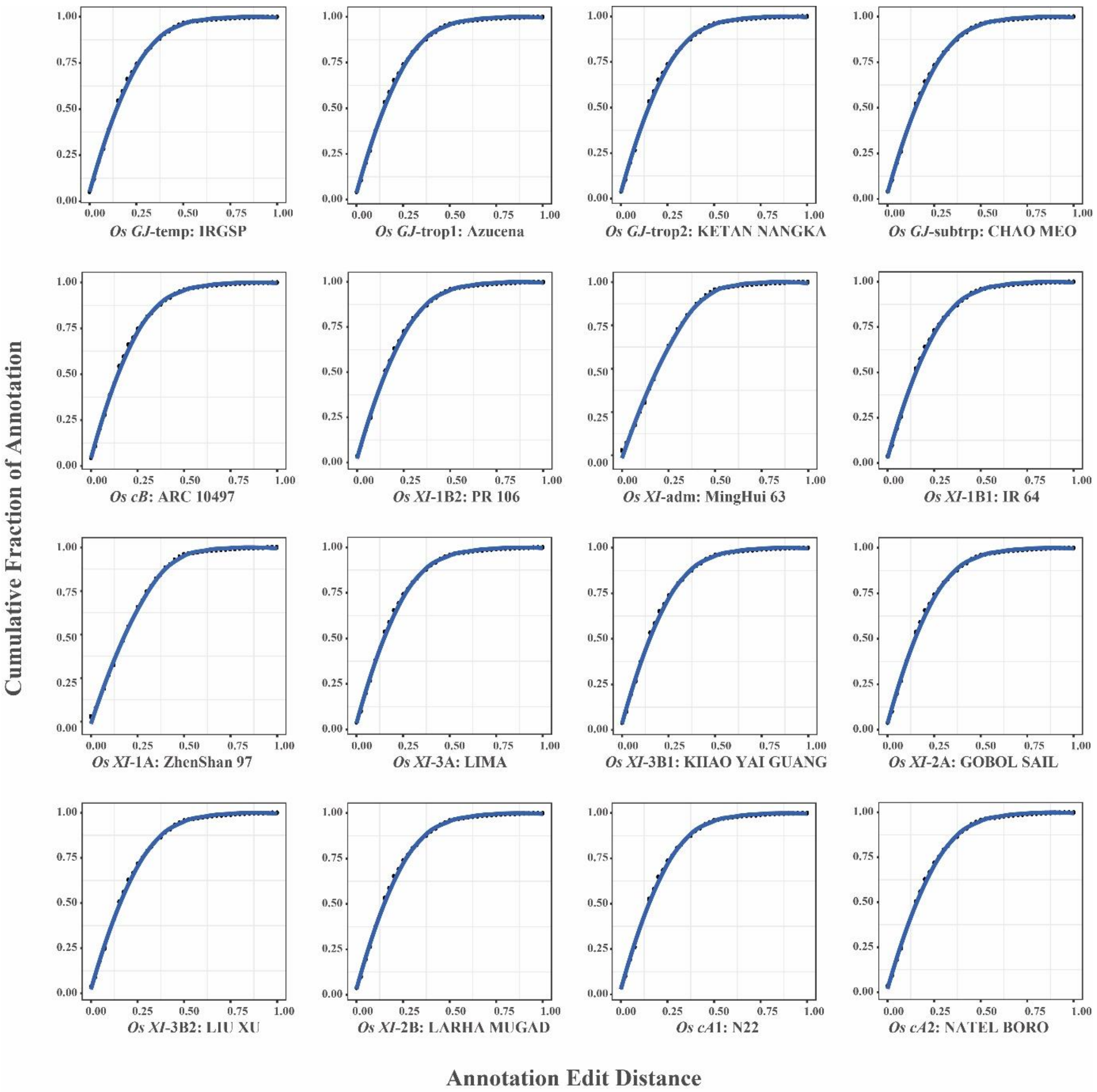

Supplementary Fig. 1. Cumulative AED distributions of 16 genomes and their annotations were plotted. Source data are provided as a Source Data file.

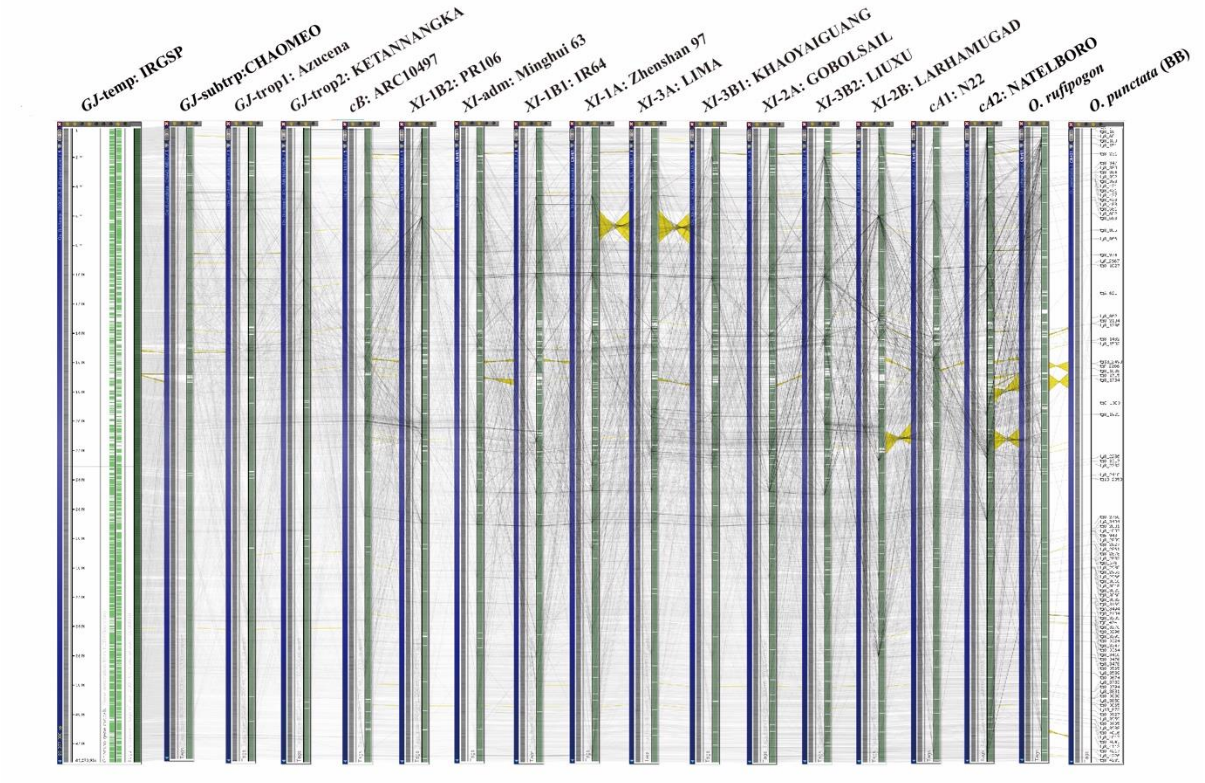

Supplementary Fig. 2. A panel showing the overall alignments of the 18-genome data packaged using chromosome 1 as an example in Persephone (https://web.persephonesoft.com/). Gray lines show the alignments of sequence tags and the yellow ribbons show the inversions.

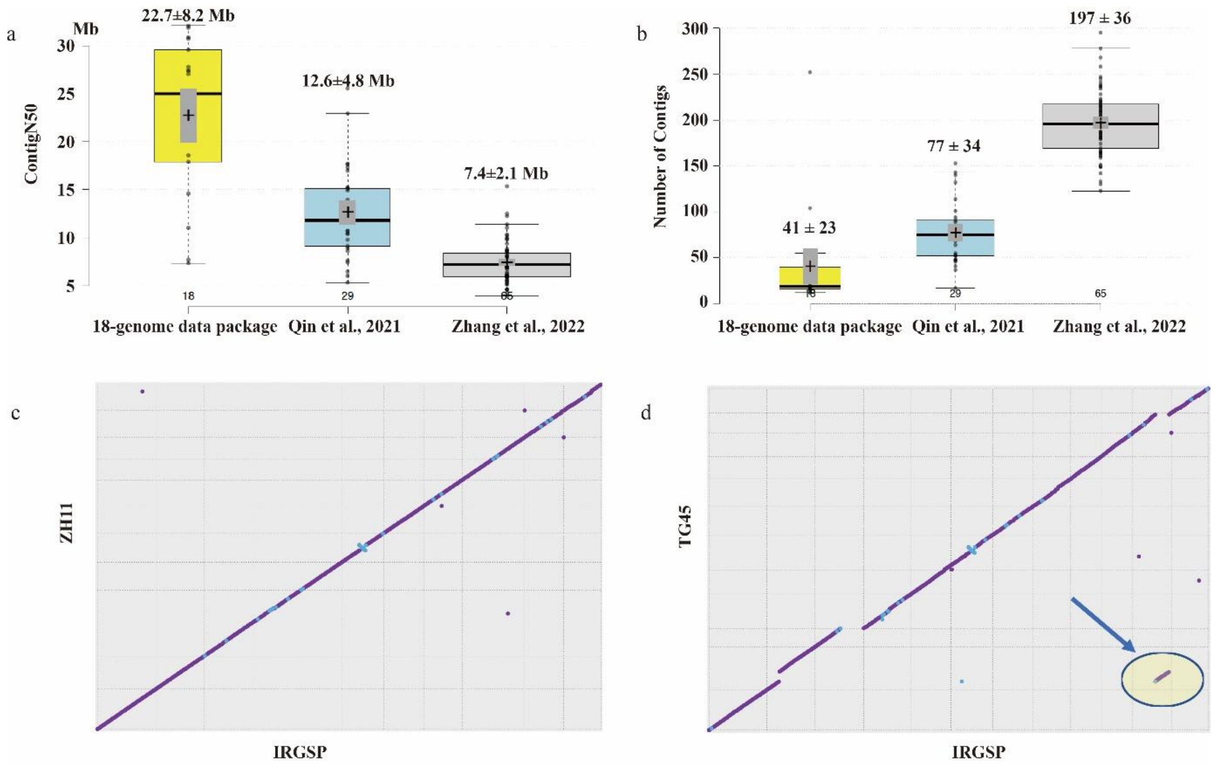

Supplementary Fig. 3. Genome quality of 112 genomes collected in this study.

a. and b. Comparisons of contigN50s and the number of contigs from the sequences of the 18-genome data package (n = 18), genomes (n = 65) from Qin et al., 2021, and genomes (n = 65) from Zhang et al., 2022, respectively. Each boxplot presents the minimum, first quartile, median, third quartile, and maximum value, and along with mean ± SD (Standard Deviation) are shown. C. and d. An example of the 75 genomes that were retained (*e.g.*, ZH11) and 28 genomes were filtered out (*e.g.*, TG45) based on genome-wide alignment validations with the IRGSP RefSeq. A comparison with TG45 detected a >5 Mb translocation from chromosome 11 to 2, however, the genome had not been validated by a third technology (*e.g.*, Bionano optical map) and was, therefore, filtered from our inversion analysis. Source data are provided as a Source Data file.

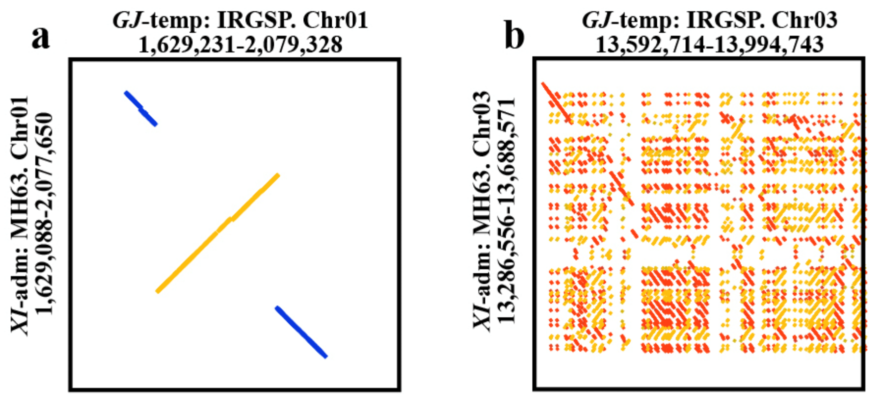

Supplementary Fig. 4. Assessment example of inversions across the 18 PSRefSeqs.
True positives show (a) clean inverted regions and breakpoints. False positives (b) are mostly due to the incorrect assessment of tandemly repeated regions.

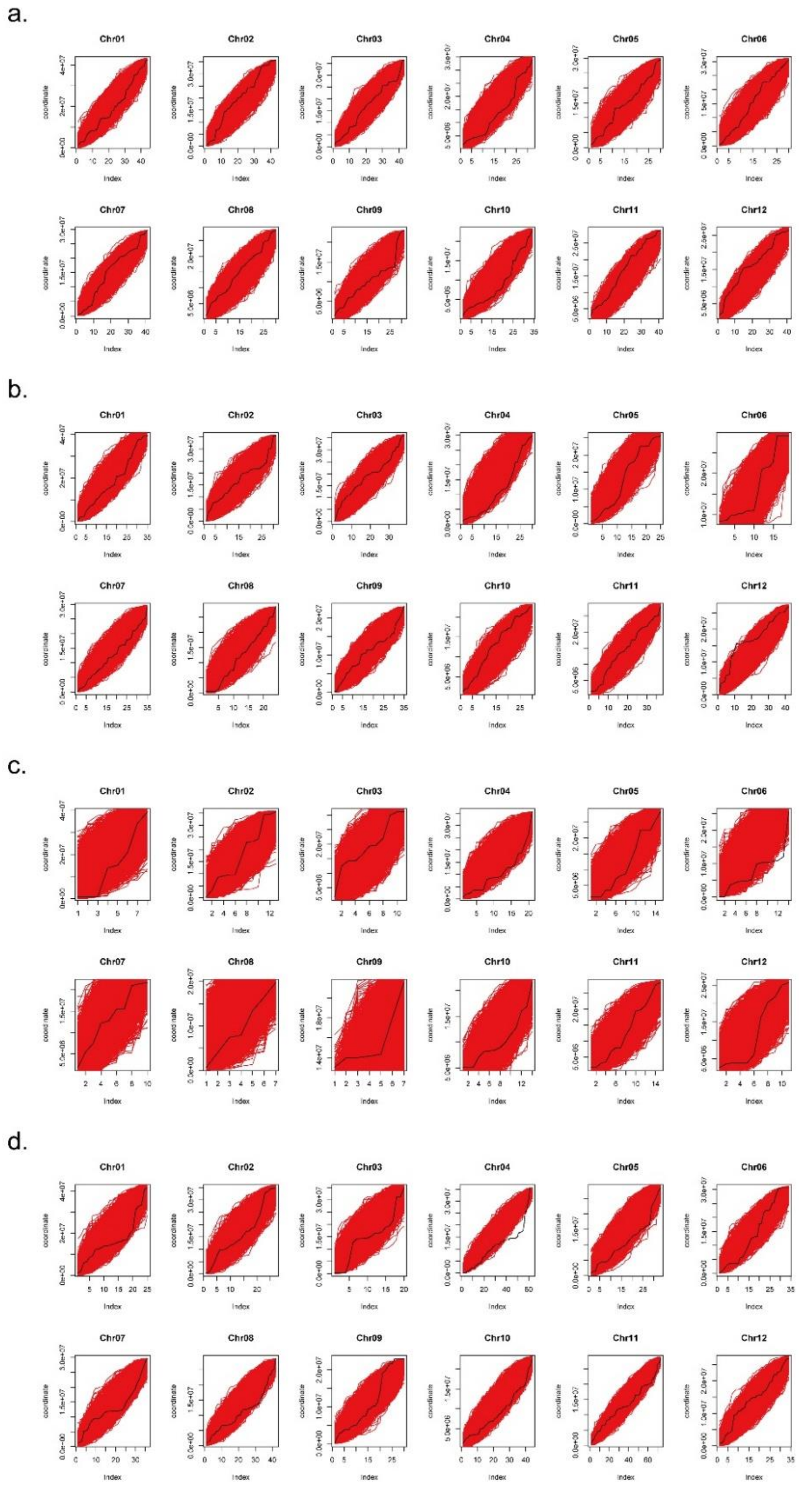

Supplementary Fig. 5. Kolmogorov-Smirnov (KS) distribution test for the pan-genome inversion index of rice.

The KS test for uniformity distribution of inversions across 12 rice chromosomes with different length categories, *i.e.*, a. < 1 Kb, b. 1 – 5 Kb, c. 5 – 10 Kb, d. > 10 Kb. The black line is the actual inversion distribution, and the red line is the 10,000th uniformly distributed simulation. Source data are provided as a Source Data file.

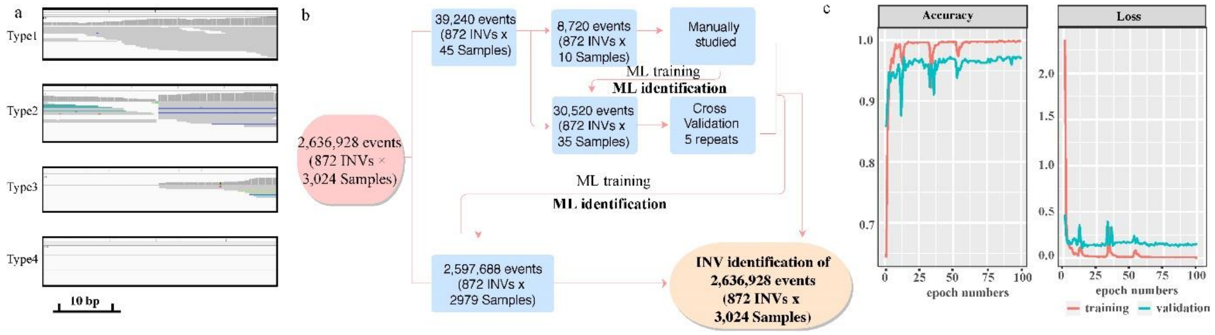

Supplementary Fig. 6. Machine learning analysis of inversion alignment patterns across the 3K-RGP data set.

a. Four different alignment patterns were observed. The Type 1 pattern shows that the inversion coordinates were covered by short read alignments of an accession but no breakpoints were observed, which means the inversion event is absent in this accession. The Type 2 pattern shows that the inversion coordinates were covered by short read alignments of an accession and the breakpoints were observed, which means the inversion event is present in this accession. The Type 3 pattern shows the that the inversion coordinates were covered by short read alignments of an accession and half of breakpoints were observed, which means the inversion event is present in this accession, but is accompanied with a deletion or another as yet to be determined, genomic rearrangement. The Type 4 pattern shows that the inversion coordinates were not covered by short read alignments of an accession, and if the breakpoints were not able to be observed, the inversion event is not present in this case. b. Workflow used for training the model with manually curated data, and predictions for unknown data. c. Accuracy and loss assessment of the model with 5 cross checks for 100 generations. Source data are provided as a Source Data file.

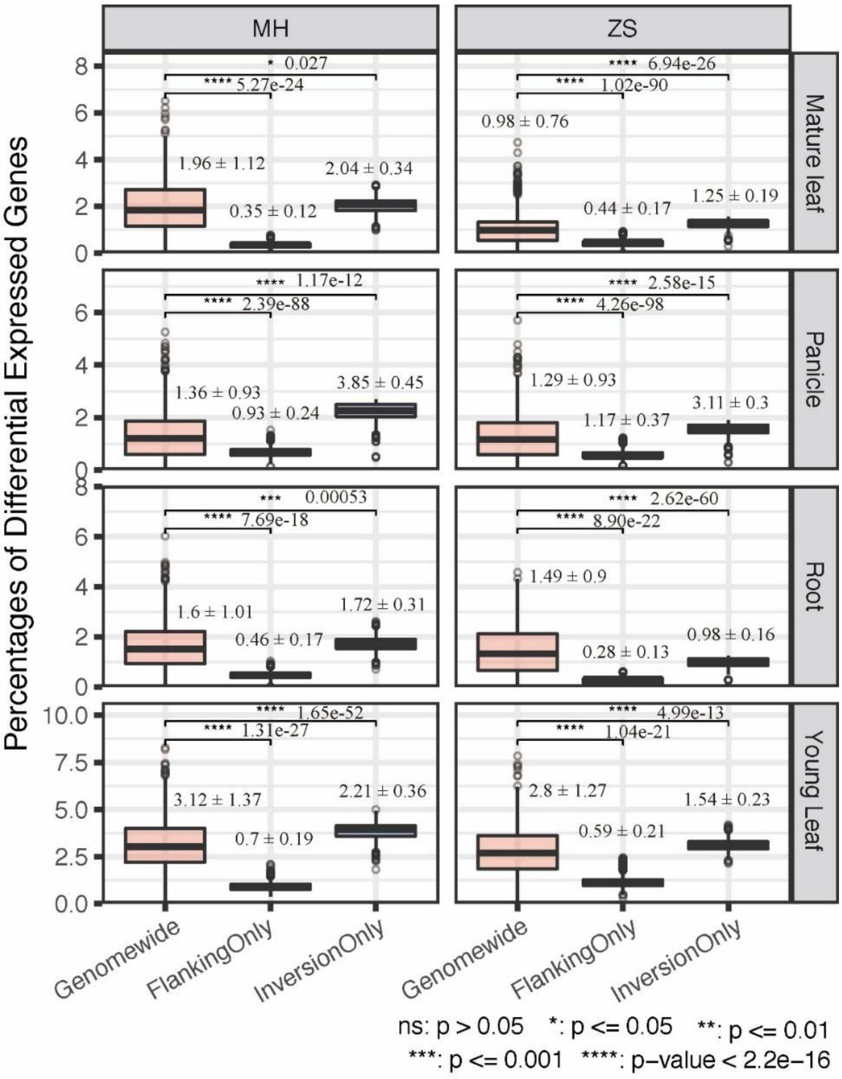

Supplementary Fig. 7. Analysis of differentially expressed genes (DEGs) located within and flanking inversions compared with controls.

DEGs analysis, from RNA-Seq dataset #2, located in inverted regions, flanking regions vs. a set of randomly selected non-inverted regions (20 Kb, n = 1,000). The DEGs from both Minghui 63 (MH) and Zhenshan 97 (ZS) were compared with IRGSP RefSeq. The percentages of DEGs of three regions (*i.e.*, within inversions, flanking regions, and resampled non-inverted 20 Kb genome-wide regions) are shown with boxplots and compared by a two tailed *t*-test. Each boxplot presents the minimum, first quartile, median, third quartile, and maximum value, and along with mean ± SD (Standard Deviation) are shown. Source data are provided as a Source Data file.

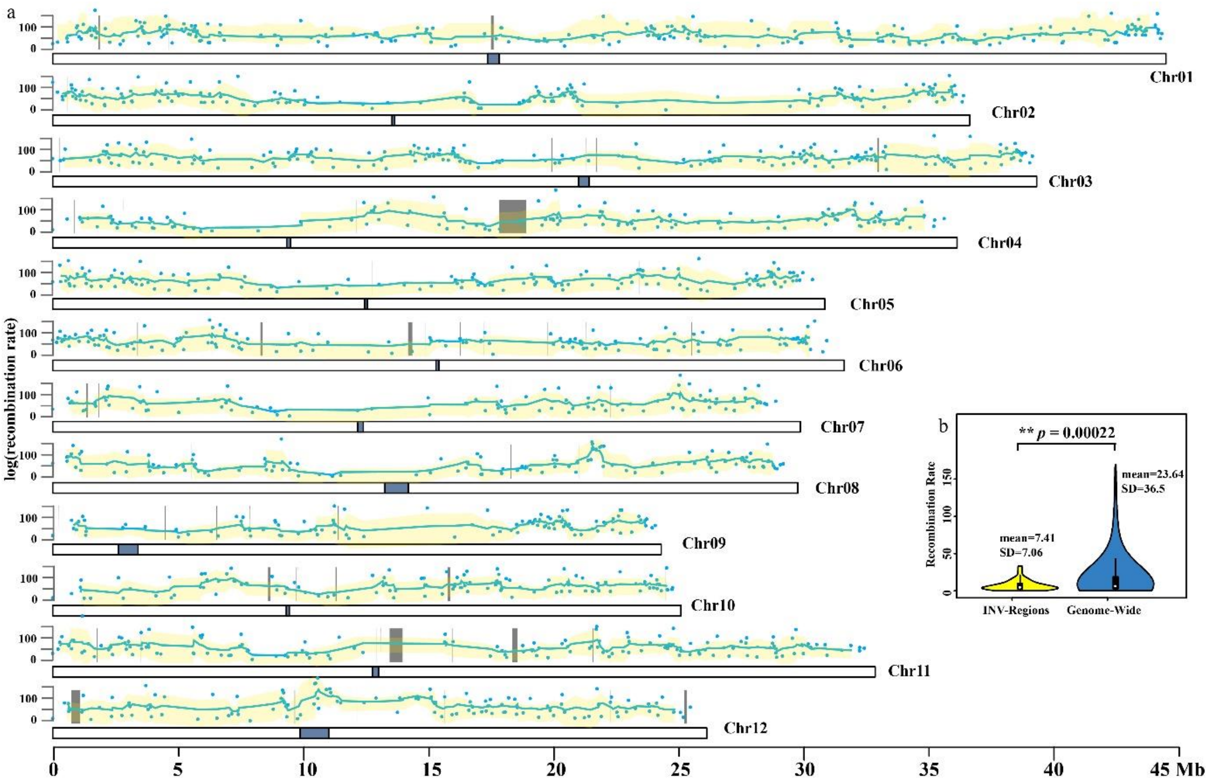

Supplementary Fig. 8. Recombination rate variation between inverted and non-inverted regions of a recombinant inbred mapping population.

a. Recombination rate of a RIL mapping population (Minghui 63 × Zhenshan 97). Dots indicate the recombination rate and gray boxes indicate inversions. Blue lines and yellow shades present the average and range of recombination rate for 100 Kb window regions along the chromosomes (rectangles). Blue boxes along the chromosomes indicate the centromeres. b. Comparison of recombination rate across inversions (n = 78) vs. genome-wide (n = 1536) (*F*-test in the ANOVA). Each violin plot presents the minimum, first quartile, median, third quartile, and maximum value, and along with mean and SD (Standard Deviation) values are shown. Source data are provided as a Source Data file.

Supplementary Table 1. PacBio Iso-Seq and RNA-Seq transcripts used for genome annotation.

Acronyms	Iso-Seq			RNA-Seq		
	Leaf	Root	Panicle	Leaf	Root	Panicle
IRGSP	19,101	26,760	31,356	40,689	42,513	35,478
CMeo	26,589	28,501	23,492	22,016	26,372	46,613
Azu	29,209	33,939	27423	23,145	25,382	39,385
KeNa	17,148	15,960	24,992	18,847	26,409	40,157
ARC	25,932	25,265	-	20,523	28,897	-
PR106	31,088	44,374	23,645	31,949	31,314	47,161
IR64	19,168	31,556	21988	23,221	30,118	43,614
Lima	32,067	26,603	-	21,724	21,322	-
KYG	31,036	43,031	-	22,317	27,844	-
GoSa	10,565	42,680	-	16,510	22,062	-
LiXu	23,628	31,519	13,159	27,391	31,386	30,855
LaMu	29,618	38,265	-	20,476	29,311	-
N22	43,709	50,872	26,818	21,821	26,431	35,043
NaBo	37,614	41,834	24,811	22,208	31,537	38,038

Supplementary Table 2. BUSCO evaluation of the *O. rufipogon* (AA) and *O. punctata* (BB) genomes compared with Asian rice pan-genome missing genes[3].

**Assembly**	**C**	**S**	**D**	**F**	**M**	**Absented genes in the 18-genome datapackage**
O. rufipogon	409 (98%)	398 (97.1%)	11 (0.8%)	3 (0.2%)	28 (1.9%)	EOG093603UO(abc), EOG093603VZ(abc), EOG0936041Q(abc), EOG093608FN(abc), EOG0936091T(abc), EOG093609SU(abc), EOG09360AW4(abc),
O. punctata	397 (97%)	390 (96.5%)	7 (0.5%)	10 (0.7%)	33 (2.3%)	EOG09360BID(abc), EOG09360CFL(abc), EOG09360CH3(abc), EOG09360MYG(abc), EOG09360TV2(abc), EOG093605AK(ab), EOG09360AWY(ab), EOG093606SW(a) EOG093601AL(a)

C: Complete BUSCOs
S: Complete and single-copy BUSCOs
D: Complete and duplicated BUSCOs
F: Fragmented BUSCOs
M: Missing BUSCOs
a: absent in all Asian and African genomes (AA genome)
b: absent in all *O. punctata* (BB genome)
c: absent in all *Zea mays*

Supplementary Table 3. Genome annotation statistics of 16 rice genomes.

Accession	No. of genes-whole genome-Chr-level	Avg. length of gene (bp)	# of cDNA/CDS/proteins	Avg. length of cDNA (bp)	Avg. length of CDSs (bp)	Avg. length of proteins (aa)
IRGSP	37,140	3,380	44,983	1,519	1,187	395
CMeo	36,601	3,430	45,044	1,548	1,194	397
Azu	36,623	3,436	44,364	1,552	1,198	398
KeNa	36,609	3,465	44,022	1,535	1,191	396
ARC	36,423	3,427	43,439	1,526	1,188	395
PR106	36,405	3,493	44,346	1,545	1,196	397
MH63	36,147	3,621	52,024	1,724	1,210	402
IR64	36,065	3,480	43,965	1,549	1,199	398
ZS97	35,686	3,675	50,681	1,719	1,224	407
Lima	36,217	3,448	43,521	1,503	1,178	391
KYG	36,212	3,445	43,155	1,511	1,184	393
GoSa	36,222	3,453	42,380	1,496	1,178	391
LiXu	36,378	3,416	43,497	1,484	1,167	388
LaMu	36,299	3,414	42,947	1,502	1,177	391
N22	36,262	3,461	43,845	1,539	1,194	397
NaBo	36,196	3,459	43,220	1,517	1,187	394
Average	36,343	3,469	44,715	1,548	1,191	396

Supplementary Table 4. Sequencing, data statistics of genomic features, and BUSCO evaluation of *de novo* assemblies for 2 new wild *Oryza* genomes, *i.e.*, *O. rufipogon* (AA) and *O. punctata* (BB).

**Variety name**	***O. rufipogon*-AA (wild Asian rice)**	***O. punctata*-BB (outgroup)**
Sequencing platform	PacBio Sequel	PacBio Sequel
Raw data (Gb)	66.09	47.94
Depth	142.88	113.50
Number of subreads (M)	3.00	5.05
Mean subread length (Kb)	22.02	9.50
N50 subread length (Kb)	37.01	16.20
Assembled Size (Mb)	462.58	422.39
Contig N50 length (Mb)	32.50	33.10
# Gaps	7	16
Complete BUSCOs	97.90%	97.00%
BioProject	PRJNA609053	PRJNA13770
BioSample	SAMN14209993	SAMN02981556
Genome Accession	JAAMOU000000000	AVCL00000000
SRP	SRP251141	SRP038011

Supplementary Table 5. The assessment of 4 workflows used to identify genomic inversions between the IRGSP-1.0 RefSeq and MH63 genome sequences.

Methods	Aligner	INV caller	Number of raw INV	Number of true INV	True INV of each workflow%	Missed in workflow4	True INV of 4 workflows %	Reference
Workflow1	NGMLR	SVIM	25	18	72.0%	1	10.0%	Heller *et al.*[24]
Workflow2	NGMLR	Sniffles	131	39	29.8%	2	21.7%	Sedlazeck *et al.*[23]
Workflow3	Minimap2	SyRI	55	33	60.0%	0	18.3%	Goel *et al.*[26]
Workflow4	Nucmer	SyRI	235	178	75.7%	NA	98.9%	Goel *et al.*[26]

Supplementary Table 6. Validation of a subset of inversions from the pan-genome inversion index using PacBio long read data.

Genome	Number of INVs	Number of INVs could be supported by PacBio LongReads
LaMu	78	75
NaBo	74	74
CMeo	35	35
MH63	77	74
Total	264	258 (97.73%)

Supplementary Table 7. Four groups and 7 categories of inversions could be identified across the pan-genome inversion index of rice.

Group	Category	*O. punctata*	*O. rufipogon*	*O. sativa*	count	Description
S					885	Inversions segregating in *O. sativa*
	S1	0	0	≥1	872	*O. sativa* specific, IRGSP-1.0 Ref-Seq has ancestral state
	S2	1	1	≥1	11	*O. sativa* specific, IRGSP-1.0 Ref-Seq has derived state
	S3	1	1	0	2	All *O. sativa* have derived state - an inversion fixed in *O. sativa*
SR					123	Inversions segregating in both *O. sativa* and *O. rufipogon*
	SR1	0	1	≥1	121	Originated before *O. sativa* and *O. rufipogon* split, or introgressions, IRGSP-1.0 RefSeq has the ances-tral state
	SR2	1	0	≥1	2	Originated before *O. sativa* and *O. rufipogon* split, or introgressions, IRGSP-1.0 RefSeq has the derived state
R					96	*O. rufipogon* specific
	R	0	1	0	96	
P					322	*O. punctata* specific or AA-fixed (ancestral state not clear)
	E	1	0	0	322	

Supplementary Table 8. Manual validation of inversions identified from machine learning.

Manual-validation	Round1	Round2	Round3	Note
Tag0	97.00%	94.69%	96.75%	Non-INV
Tag1	100.00%	99.50%	99.50%	INV
Tag3	100.00%	100.00%	99.50%	INV-pav
Tag4	99.50%	100.00%	99.50%	Can't be define

Supplementary Table 9. Validation of 2,042 predicted inversions, of the same accessions used to create the Asian rice pan-genome, collected from the 3K-RGP data set.

Length	# total	# true	# false	% true	% false
0.1-1 kb	915	14	901	1.53%	98.47%
1-25 kb	825	123	702	14.91%	85.09%
25-250 kb	532	56	476	10.53%	89.47%
250 kb	130	7	123	5.38%	94.62%
total	2,402	200	2,202	8.33%	91.67%

Supplementary references

1. Kawahara, Y. *et al.* Improvement of the *Oryza sativa* Nipponbare reference genome using next generation sequence and optical map data. *Rice* **6**, 4 (2013).
2. Zhang, J. *et al.* Extensive sequence divergence between the reference genomes of two elite indica rice varieties Zhenshan 97 and Minghui 63. *Proc. Natl. Acad. Sci. USA* **113**, E5163-E5171 (2016).
3. Zhou, Y. *et al.* A platinum standard pan-genome resource that represents the population structure of Asian rice. *Sci Data* **7**, 113 (2020).
4. Chin, C.S. *et al.* Phased diploid genome assembly with single-molecule real-time sequencing. *Nat Methods* **13**, 1050-1054 (2016).
5. Xiao, C.L. *et al.* MECAT: fast mapping, error correction, and de novo assembly for single-molecule sequencing reads. *Nat Methods* **14**, 1072-1074 (2017).
6. Koren, S. *et al.* Canu: scalable and accurate long-read assembly via adaptive k-mer weighting and repeat separation. *Genome Res* **27**, 722-736 (2017).
7. Zhang, J. *et al.* Genome puzzle master (GPM): an integrated pipeline for building and editing pseudomolecules from fragmented sequences. *Bioinformatics* **32**, 3058-3064 (2016).
8. Campbell, M.S., Holt, C., Moore, B. & Yandell, M. Genome annotation and curation using MAKER and MAKER - P. *Current Protocols in Bioinformatics* **48**, 4.11. 1-4.11. 39 (2014).
9. Qin, P. *et al.* Pan-genome analysis of 33 genetically diverse rice accessions reveals hidden genomic variations. *Cell* **184**, 3542-3558 (2021).
10. Zhang, F. *et al.* Long-read sequencing of 111 rice genomes reveals significantly larger pan-genomes. *Genome Res* **32**, 853-863 (2022).
11. Delcher, A.L., Salzberg, S.L. & Phillippy, A.M. Using MUMmer to identify similar regions in large sequence sets. https://doi.org/10.1002/0471250953.bi1003s00 (2003).
