# Supplementary material for: Pan-genome inversion index reveals evolutionary insights into the subpopulation structure of Asian rice
Source: Nat Commun. 2023 Mar 21;14:1567. doi: 10.1038/s41467-023-37004-y (PMC10030860; doi:10.1038/s41467-023-37004-y)
Supplement: Supplementary file 3 — Description of Additional Supplementary Files [file 41467_2023_37004_MOESM3_ESM.pdf]

Description of Additional Supplementary Files

File Name: Supplementary Data 1
Description: BUSCO assessments of genome annotations using both (a) transcriptome, and (b) protein model evidence.

File Name: Supplementary Data 2
Description: Metadata information, genome assembly statistics and quality control of the 112 genomes investigated in this study.

File Name: Supplementary Data 3
Description: Summary of inversions identified across the 75-genome dataset.

File Name: Supplementary Data 4
Description: Pan-genome inversions across the 75 high-quality genomes, by comparing 74 O. *sativa* accessions, and 2 close relative genomes to the IRGSP-1.0. RefSeq.

File Name: Supplementary Data 5
Description: Kolmogorov-Smirnov (KS) distribution tests for all inversions, and inversions length subsets: < 1 Kb, 1 - 5 Kb, 5 - 10 Kb, and > 10 Kb.

File Name: Supplementary Data 6
Description: Inversion hotspots in Asian rice.

File Name: Supplementary Data 7
Description: The distribution of 631 O. sativa inversions across the 3K-RGP dataset.

File Name: Supplementary Data 8
Description: Analysis of O. sativa specific inversions at the population level.

File Name: Supplementary Data 9
Description: TE annotation of 16 Oryza sativa genomes.

File Name: Supplementary Data 10
Description: 17 TEs from 4 superfamilies were observed at inversion breakpoints of 16 Asian genomes in higher amounts (> 10 in this study) relative to their presence genome-wide.

File Name: Supplementary Data 11
Description: Repeat sequence analysis at 100 randomly selected inversions.

File Name: Supplementary Data 12
Description: Comparison of transcript abundance levels for genes that were located within inversions, or at inversion breakpoints.

File Name: Supplementary Data 13
Description: Seventy-eight inversions identified between XI-adm: MH63 and XI1A: ZS97 genomes, the parents of a RIL-10 population.
Note: To identify recombination rates, we only focused on inversions > 1 Kb.

File Name: Supplementary Data 14
Description: Clusters for inversions larger than 100 Kb.

File Name: Supplementary Data 15
Description: A list of 6 inversions that were identified from the 3K-RGP data set, but were missed in the Asian rice pan-genome inversion index.
