# Supplementary material for: Pan-genome inversion index reveals evolutionary insights into the subpopulation structure of Asian rice
Source: Nat Commun. 2023 Mar 21;14:1567. doi: 10.1038/s41467-023-37004-y (PMC10030860; doi:10.1038/s41467-023-37004-y)
Supplement: Supplementary file 19 — Reporting Summary [file 41467_2023_37004_MOESM19_ESM.pdf]

nature portfolio

Corresponding author(s): Rod Wing

Last updated by author(s): Feb 20, 2023

Reporting Summary

Nature Portfolio wishes to improve the reproducibility of the work that we publish. This form provides structure for consistency and transparency in reporting. For further information on Nature Portfolio policies, see our Editorial Policies and the Editorial Policy Checklist.

Statistics

For all statistical analyses, confirm that the following items are present in the figure legend, table legend, main text, or Methods section.

n/a	Confirmed	
☐	☒	The exact sample size (*n*) for each experimental group/condition, given as a discrete number and unit of measurement
☐	☒	A statement on whether measurements were taken from distinct samples or whether the same sample was measured repeatedly
☒	☐	The statistical test(s) used AND whether they are one- or two-sided<br>*Only common tests should be described solely by name; describe more complex techniques in the Methods section.*
☒	☐	A description of all covariates tested
☐	☒	A description of any assumptions or corrections, such as tests of normality and adjustment for multiple comparisons
☐	☒	A full description of the statistical parameters including central tendency (e.g. means) or other basic estimates (e.g. regression coefficient) AND variation (e.g. standard deviation) or associated estimates of uncertainty (e.g. confidence intervals)
☐	☒	For null hypothesis testing, the test statistic (e.g. *F*, *t*, *r*) with confidence intervals, effect sizes, degrees of freedom and *P* value noted<br>*Give P values as exact values whenever suitable.*
☒	☐	For Bayesian analysis, information on the choice of priors and Markov chain Monte Carlo settings
☒	☐	For hierarchical and complex designs, identification of the appropriate level for tests and full reporting of outcomes
☒	☐	Estimates of effect sizes (e.g. Cohen's *d*, Pearson's *r*), indicating how they were calculated

Our web collection on statistics for biologists contains articles on many of the points above.

Software and code

Policy information about availability of computer code

Data collection	The genome sequences were collected by PacBio and Illumina sequencers.
Data analysis	FALCON (v0.5), MECAT2 (20190314), Canu (v1.5), GPM (v1.0), SMRTLink (v6.0), BUSCO (v4.0), MAKER-P (v3.01.03), FGENESH (v8.0.0), SNAP (v0.15.7), RepeatMasker (v4.0.8), StringTie (v1.3.4a), Cufflinks (v2.2.1), STAR (v2.5.3a), SAMTools (v1.9), Cd-hit (v4.6), SUPPA2 (v2), PASA (v2.4.1), InterProScan (v5.38-76.0), TRaCE (https://github.com/warelab/TRaCE), BLAST (2.2.5), EDTA (v1.7.4), NGMLR (v0.2.7), SVIM (v1.1.0), Sniffles (v1.0.7), Minimap2 (2.15), SyRI (v1.4), MUMmer (v4), SURVIVOR (v1.0.7), HISAT2 (version 2.2.1), edgeR (3.1.0). The custom code is available at https://yongzhou2019.github.io/Rice-Population-Reference-Panel/software/.

For manuscripts utilizing custom algorithms or software that are central to the research but not yet described in published literature, software must be made available to editors and reviewers. We strongly encourage code deposition in a community repository (e.g. GitHub). See the Nature Portfolio guidelines for submitting code & software for further information.

Data

Policy information about availability of data

All manuscripts must include a data availability statement. This statement should provide the following information, where applicable:

- Accession codes, unique identifiers, or web links for publicly available datasets
- A description of any restrictions on data availability
- For clinical datasets or third party data, please ensure that the statement adheres to our policy

All data are available as supplementary tables.
All sequencing data has been submitted to the NCBI. The assembly data of IRGSP-1.0/NIPPONBARE (GCA_001433935.1 [https://www.ncbi.nlm.nih.gov/assembly/GCF_001433935.1/]), CHAO MEO::IRGC 80273-1 (GCA_009831315.1 [https://www.ncbi.nlm.nih.gov/assembly/GCA_009831315.1/]), Azucena (GCA_009830595.1 [https://www.ncbi.nlm.nih.gov/assembly/GCA_009830595.1/]), KETAN NANGKA::IRGC 19961-2 (GCA_009831275.1 [https://www.ncbi.nlm.nih.gov/assembly/GCA_009831275.1/]), ARC 10497::IRGC 12485-1 (GCA_009831255.1 [https://www.ncbi.nlm.nih.gov/assembly/GCA_009831255.1/]), PR 106::IRGC 53418-1 (GCA_009831045.1 [https://www.ncbi.nlm.nih.gov/assembly/GCA_009831045.1/]), Minghui 63 (GCA_001623365.2 [https://www.ncbi.nlm.nih.gov/assembly/GCA_001623365.2/]), IR 64 (GCA_009914875.1 [https://www.ncbi.nlm.nih.gov/assembly/GCA_009914875.1/]), Zhenshan 97 (GCA_001623345.2 [https://www.ncbi.nlm.nih.gov/assembly/GCA_001623345.2/]), LIMA::IRGC 81487-1 (GCA_009829395.1 [https://www.ncbi.nlm.nih.gov/assembly/GCA_009829395.1/]), KHAO YAI GUANG::IRGC 65972-1 (GCA_009831295.1 [https://www.ncbi.nlm.nih.gov/assembly/GCA_009831295.1/]), GOBOL SAIL (BALAM)::IRGC 26624-2 (GCA_009831025.1 [https://www.ncbi.nlm.nih.gov/assembly/GCA_009831025.1/]), LIU XU::IRGC 109232-1 (GCA_009829375.1 [https://www.ncbi.nlm.nih.gov/assembly/GCA_009829375.1/]), LARHA MUGAD::IRGC 52339-1 (GCA_009831355.1 [https://www.ncbi.nlm.nih.gov/assembly/GCA_009831355.1/]), N22 (N 22::IRGC 19379-1) (GCA_001952365.2 [https://www.ncbi.nlm.nih.gov/assembly/GCA_001952365.2/]), NATEL BORO::IRGC 34749-1 (GCA_009831335.1 [https://www.ncbi.nlm.nih.gov/assembly/GCA_009831335.1/]), O. rufipogon PNG91-7::IRGC 106523-1 (GCA_023541355.1 [https://www.ncbi.nlm.nih.gov/assembly/GCA_023541355.1/]), and O. punctata:: IRGC 105690 (GCA_000573905.2 [https://www.ncbi.nlm.nih.gov/assembly/GCA_000573905.2/]) were deposited in NCBI under GeneBank. The raw sequencing data of O. rufipogon and O. punctata generated in this study were deposited in NCBI under BioProject PRJNA609053 [https://www.ncbi.nlm.nih.gov/bioproject/?term=PRJNA609053] and PRJNA13770 [https://www.ncbi.nlm.nih.gov/bioproject/?term=PRJNA13770], respectively. PacBio Iso-Seq data for all accessions in this study was deposited in NCBI under BioProject PRJNA760839 [https://www.ncbi.nlm.nih.gov/bioproject/PRJNA760839]. The RNA-Seq dataset#1 was deposited in NCBI under BioProject PRJNA659864 [https://www.ncbi.nlm.nih.gov/bioproject/?term=PRJNA659864]. RNA-Seq dataset#2 was retrieved from NCBI BioProject PRJNA597070 [https://www.ncbi.nlm.nih.gov/bioproject/PRJNA597070]. The details are also listed in Table1 and the Rice Population Reference Panel (https://yongzhou2019.github.io/Rice-Population-Reference-Panel/data/).
Source data are provided with this paper.

Human research participants

Policy information about studies involving human research participants and Sex and Gender in Research.

Reporting on sex and gender	NA
Population characteristics	NA
Recruitment	NA
Ethics oversight	NA

Note that full information on the approval of the study protocol must also be provided in the manuscript.

Field-specific reporting

Please select the one below that is the best fit for your research. If you are not sure, read the appropriate sections before making your selection.

☒ Life sciences ☐ Behavioural & social sciences ☐ Ecological, evolutionary & environmental sciences

For a reference copy of the document with all sections, see nature.com/documents/nr-reporting-summary-flat.pdf

Life sciences study design

All studies must disclose on these points even when the disclosure is negative.

Sample size	The sample size was determinated based on a permutation test with re-sampling that showed the reasonable number of samples had been used.
Data exclusions	The genome assemblies with the large translocation (>5M) that could not be validated were removed
Replication	We confirmed the ability to replicate all code of this study.
Randomization	Randomization does not directly apply to the genome sequencing and assembly.

Blinding	No analyses required being blind to groups

Reporting for specific materials, systems and methods

We require information from authors about some types of materials, experimental systems and methods used in many studies. Here, indicate whether each material, system or method listed is relevant to your study. If you are not sure if a list item applies to your research, read the appropriate section before selecting a response.

Materials & experimental systems

n/a	Involved in the study
☒	☐ Antibodies
☒	☐ Eukaryotic cell lines
☒	☐ Palaeontology and archaeology
☒	☐ Animals and other organisms
☒	☐ Clinical data
☒	☐ Dual use research of concern

Methods

n/a	Involved in the study
☒	☐ ChIP-seq
☒	☐ Flow cytometry
☒	☐ MRI-based neuroimaging
